# High relaxivity Gd^3+^-based organic nanoparticles for efficient magnetic resonance angiography

**DOI:** 10.1186/s12951-022-01363-3

**Published:** 2022-03-31

**Authors:** Zhuang Liu, Menglong Zhao, Han Wang, Zi Fu, Hongbo Gao, Weijun Peng, Dalong Ni, Wei Tang, Yajia Gu

**Affiliations:** 1 Department of Radiology, Fudan University Shanghai Cancer Center, Shanghai, 200032 China; 2 Department of Oncology, Shanghai Medical College, Fudan University, Shanghai, 200032 China; 3 Department of Radiology, Zhongshan Hospital, Fudan University and Shanghai Institute of Medical Imaging, Shanghai, 200032 China; 4 Department of Orthopaedics, Shanghai Key Laboratory for Prevention and Treatment of Bone and Joint Diseases, Shanghai Institute of Traumatology and Orthopaedics, Ruijin Hospital, Shanghai Jiao Tong University School of Medicine, Shanghai, 200025 China; 5 Department of Radiation Oncology, Huadong Hospital Affiliated to Fudan University, Shanghai, 200040 China

**Keywords:** MR angiography, Contrast agent, Vascular imaging, Magnetic nanoparticles, Gd-chelate

## Abstract

Contrast-enhanced MR angiography (MRA) is a critical technique for vascular imaging. Nevertheless, the efficacy of MRA is often limited by the low rate of relaxation, short blood-circulation time, and metal ion-released potential long-term toxicity of clinical available Gd-based contrast agents. In this work, we report a facile and efficient strategy to achieve Gd-chelated organic nanoparticles with high relaxivity for *T*_*1*_-weighted MRA imaging. The Gd-chelated PEG-TCPP nanoparticles (GPT NPs) have been engineered composite structured consisting of Gd-chelated TCPP and PEG. The spherical structure of TCPP offers more chemical sites for Gd^3+^ coordination to improve the relaxivity and avoid leakage of the Gd^3+^ ions. The synthesized GPT NPs exhibit a high relaxation rate of 35.76 mM^− 1^ s^− 1^ at 3.0 T, which is higher than the rates for most reported MR contrast agents. Therefore, GPT NPs can be used for MRA with much stronger vascular signals, longer circulation time, and high-resolution arterial vascular visualization than those using clinical MR contrast agents at the same dose. This work may make the *T*_*1*_ MRI contrast agents for high-resolution angiography possible and offer a new candidate for preclinical and clinical applications of MR vascular imaging and vascular disease diagnosis.

**Graphical Abstract:**

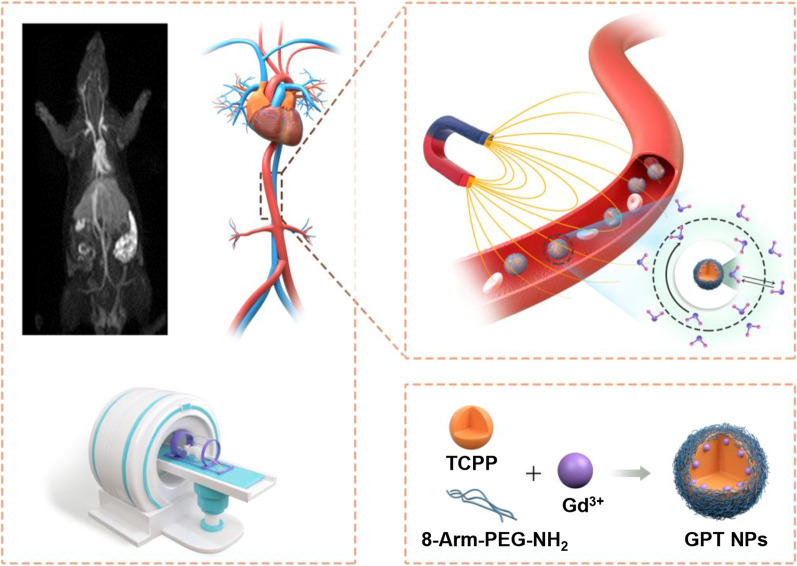

**Supplementary Information:**

The online version contains supplementary material available at 10.1186/s12951-022-01363-3.

## Background

 Cerebrovascular and cardiovascular diseases remain the leading causes of morbidity and mortality worldwide [1]. Therefore, the early and accurate diagnosis of these diseases is of vital importance for clinical procedures [2–4]. Digital subtraction angiography (DSA), the gold standard for diagnosing cerebrovascular and cardiovascular diseases, is invasive and exposes patients to ionizing radiation [5]. Recently, computed tomography angiography (CTA) has been demonstrated as a reliable alternative to enhance the contrast-to-noise ratio (CNR) without invasion. Because of the low intrinsic intensity, CT requires contrast agents to achieve increased signal-to-noise ratio (SNR) for accurate diagnosis [6]. However, the administration of iodinated contrast material with a high dose might increase acute hypersensitivity reactions with an occurrence rate of about 0.3–3%, such as acute spontaneous or infection-induced urticaria, even severe respiratory and/or cardiovascular organ systems anaphylaxis [7, 8].

Compared to conventional DSA and CTA, three-dimensional contrast-enhanced MR angiography (3D CE MRA) has attracted increasing research interest due to their superiority, such as non-invasiveness, non-ionizing radiation, 3D reconstruction, and low incidence of hypersensitivity reactions, which make it more suitable for repeat examinations [9–14]. However, evaluation of vascular imaging with MRA encounters several challenges. First, the commercial gadolinium (Gd)-based MRA contrast agents are mainly extracellular agents, such as *Omniscan* (gadodiamide, Gd-DTPA-BMA) and *Magnevist* (gadopentetate dimeglumine, Gd-DTPA), which possess a relatively short blood-circulation half-life time with several minutes [15]. Second, following intravenous administration, these gadolinium (Gd)-based MRA contrast agents rapidly diffuse across vessels into the interstitial space, showing a shortened time window for imaging blood vessels and overlapping with enhancing tissue [16]. Last, these contrast agents still suffer from a low rate of relaxation, hindering their clinical use in vascular imaging [17]. Hence it is necessary to develop a novel MRA contrast agent with long circulation in the blood and strong *T*_*1*_ contrast.

**Scheme 1:**
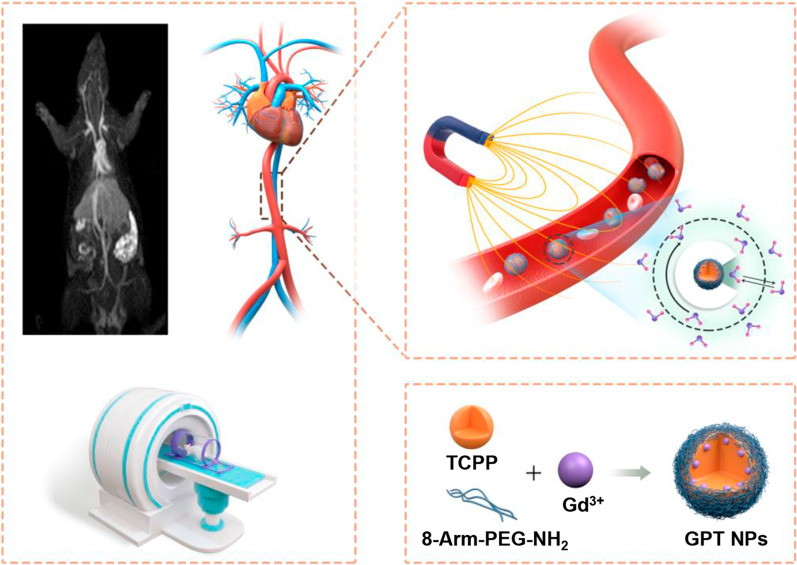
A schematic diagram illustrates the facile synthesis of Gd-chelated TCPP PEG nanoparticles (GPT NPs) and their biomedical application as contrast agents for MR angiography (MRA)

In this work, we have designed and fabricated a composite nanoplatform consisting of Gd-chelated tetrakis (4-carboxyphenyl)-porphyrin (TCPP) and 8-arm-amine-polyethylene glycol (PEG) (Gd-chelated PEG-TCPP nanoparticles, GPT NPs) with long circulation time in blood and strong *T*_*1*_ contrast for improving the sensitivity of vascular imaging. As shown in Scheme 1, the GPT NPs were facilely synthesized via robust and mild synthesis by complexing TCPP with PEG in DMSO. The center of TCPP offered a stable environment for Gd^3+^ coordination to improve the paramagnetic property and the outer surface PEG endows GPT NPs with prolonged blood circulation time and excellent biocompatibility. These GPT NPs exhibited an extremely high relaxation rate with *r*_1_ value reaching 35.76 mM^− 1^ s^− 1^ at 3.0 T. In vivo MRA imaging of rats and rabbits found that these GPT NPs showed longer circulation time and high-resolution arterial visualization than the clinically used Gd-based contrast agents, showing purely arterial image and negligible overlap with enhancing veins and tissue. Importantly, the high-resolution imaging of the heart was realized with the synthesized GPT NPs, clearly showing the detailed structures of the heart. GPT NPs can be used for MRA with much stronger vascular signals, longer circulation time, and high-resolution arterial vascular visualization than those using clinical MR contrast agents at the same dose. Our results may provide new perspectives to address the *T*_*1*_ MRI contrast agents for the high-resolution angiography and offer a new candidate for preclinical and clinical applications of MR vascular imaging and vascular disease diagnosis.

## Materials and methods

### Chemicals and reagents

Tetrakis (4-carboxyphenyl)-porphyrin (TCPP) and 3-(3-Dimethylaminopropyl)-1-Ethylcarbodiimide Hydrochloride (EDC) were purchased from Tokyo Chemical Industry (TCI). 8-arm-amine-polyethylene glycol (8-arm-PEG_4k_-NH_2_) was obtained from Shanghai Yare Biotech. Dimethyl sulfoxide (DMSO) was purchased from Sinopharm Chemical Reagents. Gadolinium trichloride hexahydrate (GdCl_3_·6H_2_O), phosphate buffer solution (PBS), DMEM high glucose, trypsin-EDTA, penicillin-streptomycin (P/S) solution, fetal bovine serum (FBS), and penicillin streptomycin were purchased from Shanghai Titan Technology (ADAMAS). Cell counting Kit-8 (CCK-8) was purchased from Beyotime Biotech. PD-10 columns were purchased from Cytiva. All reagents were used without any purification.

### Characterization

Transmission electron microscopy (TEM) and X-ray energy dispersive spectroscopy (EDS) was performed on a field emission transmission electron microscope (TALOS F200X, US). Dynamic light scattering (DLS) measurement was acquired on Nano-Zetasizer (Nano ZS90, UK). X-ray photoelectron spectroscopy (XPS) was performed on an ESCAlab250 X-ray photoelectron spectroscopy (Thermal Scientific). Fluorescence spectroscopy was acquired on FLS980 (Edinburgh, UK). The concentrations of samples were determined by an inductively coupled plasma optical emission spectrometer (ICP-OES, Thermo Fisher, US). In vitro MR imaging evaluations were conducted on a clinical MRI scanner (GE SIGNA Architect 3.0 T). In vivo MR angiography was performed on a clinical MRI scanner (MAGNETOM Prisma 3.0 T).

### Synthesis of PEG-TCPP nanoparticles

The PEG-TCPP nanoparticles were synthesized using a reported approach [18]. In a typical procedure, tetrakis (4-carboxyphenyl)-porphyrin (TCPP), 8-arm-amine-polyethylene glycol (PEG) (molecular weight 2 K), and 3-(3-Dimethylaminopropyl)-1-Ethylcarbodiimide Hydrochloride (EDC) (the ratio of mTCPP: 8Arms-PEG: EDC was 1:2:4) were mixed in 50 mL dimethyl sulfoxide (DMSO) and the reaction was stirred in the dark for 24 h at room temperature under argon gas protection. The resulting PEG-TCPP nanoparticles were collected into a dialysis bag (7 KD cut-off) and dialyzed against aqueous solution under sustained stirring at room temperature for 14 days.

### Synthesis of Gd-chelated PEG-TCPP nanoparticles

Gd-chelated PEG-TCPP nanoparticles (GPT NPs) were synthesized by a one-step method. Typically, the above prepared PEG-TCPP nanoparticles were adjusted to pH 5.5 using sodium acetate solution (10 mL). Then, 2.5 mL of GdCl_3_·6H_2_O (0.5 mM) was added into the solution and allowed to react at 70 °C for 4 h to obtain Gd-chelated PEG-TCPP nanoparticles via the chelation between Gd^3+^ and TCPP. The resulting Gd-chelated PEG-TCPP nanoparticles were purified by a PD-10 columns using PBS sodium acetate solution (pH = 5.5) as the mobile phase.

For dynamic evaluation of Gd^3+^ leakage, GPT NPs and *Omniscan* aqueous solution was put in a dialysis bag (molecular cutoff  weight of 5 KD), which was further soaked in different buffer solutions (Water, PBS, SBF, Saline, and DMEM). The Gd^3+^ ions were acquired at given time intervals.

### In vitro cytotoxicity experiments

Pancreatic ductal epithelial cells (PDEC) and human pancreatic cancer PANC-1 cells were originally obtained from the Shanghai Institute of Cells, Chinese Academy of Sciences and cultured under recommended conditions. All cells were maintained in media (DMEM) supplemented with 10% fetal bovine serum (FBS) and 1% penicillin/streptomycin (P/S). Then these cells were seeded into 96-well plates at a density of 10^4^ cells per well to adhere for 12 h. Then, the culture medium was changed to fresh culture solution with varying concentrations of GPT NPs and *Omniscan* at elevated concentrations (Gd concentration: 0, 0.125, 0.25, 0.5, 1 mM) for 24 or 48 h. The relative cell viabilities were measured using the standard Cell Counting Kit-8 assay (CCK-8).

### In vivo toxicity experiments

All animal studies were conducted with the approval and according to the recommendations established by the administrative committee of laboratory animals of Fudan University (approval numbers 2020 JS-161). Healthy male BALB/c mice (4 weeks) were purchased from Shanghai SLAC Laboratory Animal Co. Ltd and maintained in a specific pathogen-free (SPF) environment during the experiments. Sixteen BALB/c mice were randomly divided into four groups (n = 4) and intravenously administered with GPT NPs at elevated doses (0, 5, 10, 20 mg kg^− 1^). During the 30 days’ period, the body weight of mice was measured every other day. Hematological and histological analyses were performed on the 30th day after intravenous injection. Liver function indexes were measured by the serum levels of alanine aminotransferase (ALT), aspartate aminotransferase (AST), alkaline phosphatase (ALP). Kidney function indexes were measured by the serum levels of blood urea nitrogen (BUN) and creatinine (CR). Hematological white blood cells analyses were measured by the serum levels of white blood cells (WBC), lymphocyte (LYM), monocyte (MON), and granulocyte (GRAN). Hematological platelets analyses were measured by the serum levels of platelets count (PLT), mean platelets volume (MPV), platelets ratio (PCT), and platelet-large cell rate (P-LCR). Hemoglobin and red blood cells analyses were measured by the serum levels of red blood cells (RBC), hemoglobin (HGB), hematocrit (HCT), mean corpuscular volume (MCV), mean corpuscular hemoglobin (MCH), mean corpuscular hemoglobin concentration (MCHC), red blood cell volume distribution width (RDW) and red blood cell volume distribution width standard deviation (RDW-SD).

### In vivo blood assays

For in vivo blood circulation experiments, the health rats were intravenously injected with GPT NPs (0.1 mmol kg^− 1^ of Gd, n = 3). 15 µL of blood was collected at a given time (5, 10, 30 min, 1, 2, 4, 8 and 24 h) after injection. The blood sample was dispersed into 1 mL sterile saline with nitric acid to resolve GPT NPs for the determination of Gd concentration of the GPT NPs. The concentration of Gd element was measured by ICP-OES. In vivo blood terminal half-life of GPT NPs was determined by a double-component pharmacokinetic model.

### In vivo blood free Gd^3+^ leakage from GPT NPs

For in vivo free Gd^3+^ leakage experiments, the health rats were intravenously injected with GPT NPs (0.1 mmol kg^− 1^ of Gd, n = 3). 15 µL of blood was collected at a given time (5, 10, 30 min, 1, 2, 4, 8 and 24 h) after injection. The blood sample was dispersed into 1 mL sterile saline and was put in a dialysis bag (molecular cutoff weight of 5 KD), which was further measured by ICP-OES.

### In vitro T_1_-weighted MR imaging experiments

For in vitro MR relaxation text, GPT NPs and *Omniscan* were diluted in Eppendorf tubes (2 mL volume) at various Gd concentration (0.006–0.1 mM) concentrations. *T*_*1*_ map sequence: repetition time (TR) = 1000, 2000, 3000, 4000 ms; echo time (TE) = 8 ms; slice thickness = 2 mm; space = 0.5 mm; field of view (FOV) = 18; phase FOV = 0.8; freq × phase = 256 × 192; number excitations (NEX) = 2; echo train length (ETL) = 3. MR images were transferred to ADW 4.6 workstation (GE Healthcare, US). The mean *T*_*1*_ values of each sample were measured by regions-of-interest (ROI) on the *T*_*1*_-mapping post-processing software and the relaxation rate *r*_*1*_ was determined according to the linear plot of *1/T*_*1*_ versus Gd concentration.

### Animals for the MRI experiments

Sprague-Dawley rats and New Zealand rabbits were purchased from Shanghai SLAC Laboratory Animal Co. Ltd. All animal studies were conducted under protocols approved by the Fudan University Institutional Animal Care and Use Committee (approval numbers 2020 JS-161). For MRI scanning, rats were anesthetized with tiletamine hydrochloride and zolazepam hydrochloride (Zoletil 50) at the dosage of 50 mg kg^− 1^ intramuscularly. New Zealand rabbits were anesthetized with tiletamine hydrochloride and zolazepam hydrochloride (Zoletil 50) at the dosage of 10 mg kg^− 1^ intramuscularly. GPT NPs and *Omniscan* were bolus injected intravenously through the tail vein (for rats) or ear vein (for rabbits).

### 3D CE MRA

The GPT NPs and *Omniscan* (0.1 mmol kg^− 1^ of Gd) was injected into the rats via tail vein before MRA. Three-dimensional contrast-enhanced MR angiography (3D CE MRA) images of rats were acquired MRA sequences with the following parameters: TR = 3.6 ms; TE = 1.3 ms; slice thickness = 1.0 mm; FOV = 134 × 238 mm; acquisition number = 1; number of averages = 16; and total acquisition time = ~ 5 min. 3D CE MRA images of rabbits were acquired MRA sequences with the following parameters: TR = 3.4 ms; TE = 1.2 ms; slice thickness = 1.0 mm; FOV = 174 × 399 mm; acquisition number = 1; number of averages = 16. Using this sequence, single slice images at different phases could be obtained. Then, 3D MIP images were acquired by the Siemens post-processing software manually.

### T_1_-weighted MRI

The male BALB/c nude mice (4 weeks) were purchased from Shanghai SLAC Laboratory Animal Co. Ltd. PANC-1 pancreatic tumor allograft was established by subcutaneous injection of 10^7^ PANC-1 pancreatic cells in PBS (200 µL) into the right hind limb of male BALB/c nude mouse. After the tumor growth for about 14 days, the GPT NPs and *Omniscan* (0.1 mmol kg^− 1^ of Gd) was injected into the mice via tail vein. The scan sequence was *T*_*1*_-weighted Fast-recovery spin-echo (FSE) with the parameters were described as follows: TR = 480 ms; TE = 14 ms; slice thickness = 2.0 mm; FOV = 134 × 134 mm; acquisition number = 1; number of averages = 16. Signal intensities of the tumor site were measured by regions-of-interest (ROI) on the *T*_*1*_ FSE imaging.

## Results and discussion

### Preparation and characterization of Gd-chelated PEG-TCPP nanoparticles

**Fig. 1:**
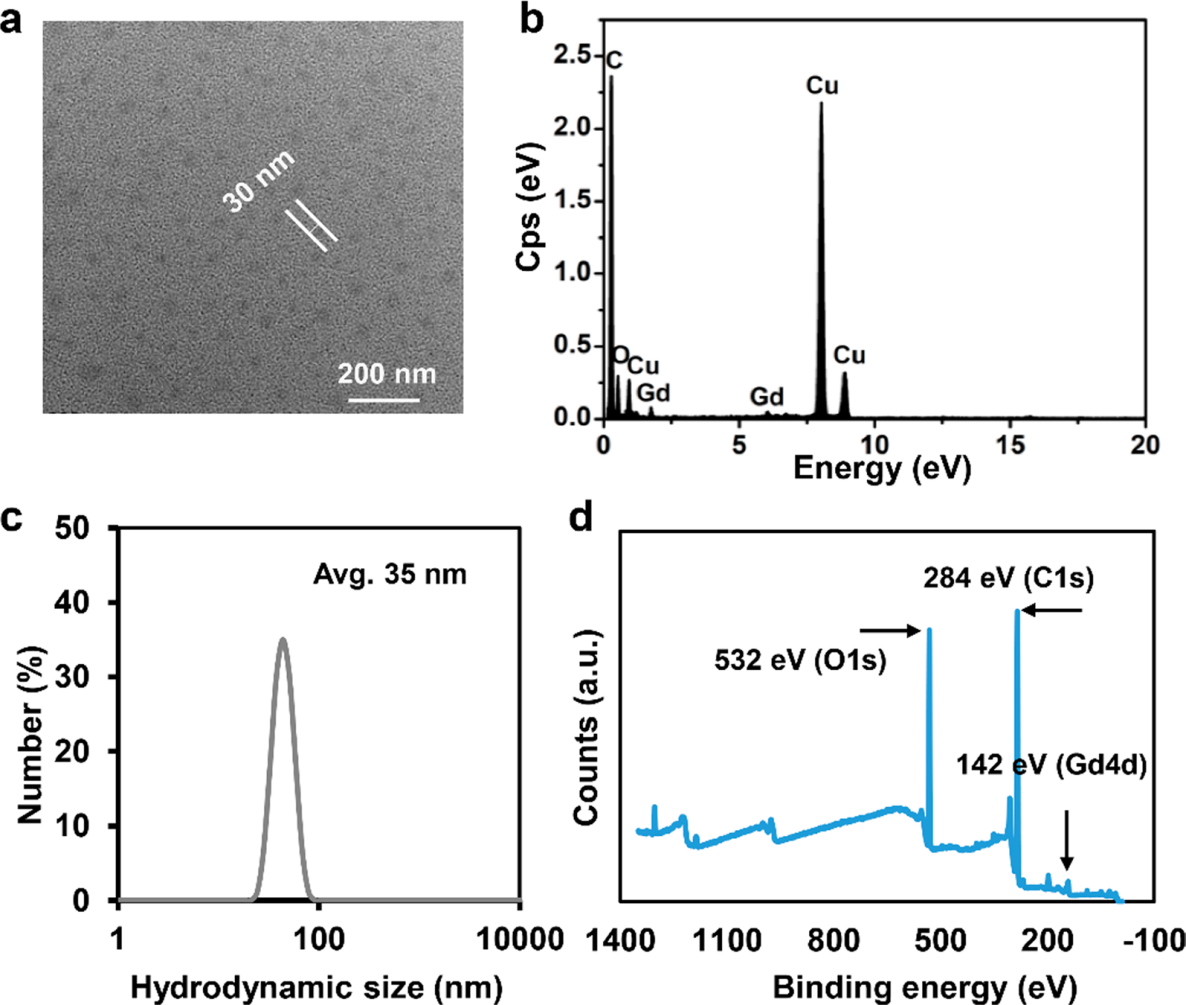
Morphology and characterization of as-synthesized GPT NPs. **a** TEM image and **b** X-ray energy dispersive spectroscopy (EDS) of GPT NPs. **c** Dynamic light scattering (DLS) curves of GPT NPs in aqueous solution. **d** XPS spectra of GPT NPs

The GPT nanoparticles were synthesized by complexing TCPP with 8-arm-PEG_4k_-NH_2_ and induced by dimethyl sulfoxide (DMSO) at room temperature under argon gas protection via a previously reported method [18]. Then, the introduced Gd^3+^ was chelated to the center of TCPP to endow the GPT NPs with paramagnetic property, which could act as the contrast agents for *T*_*1*_-weighted MRA. The as-synthesized GPT NPs were further purified by PD-10 columns several times to decrease particle aggregation and residual Gd^3+^ ions in the nanoparticle suspensions. Transmission electron microscopy (TEM) images indicate that as-prepared GPT NPs have well-fabricated spherical structure (Fig. 1a and Additional file 1: Fig. S1) with the average size of 30 nm. X-ray energy dispersive spectroscopy (EDS) shown in Fig. 1b verifies the existence of Gd elements in GPT NPs. The average hydrodynamic diameter of GPT NPs suspended in water is 35 nm as determined by dynamic light scattering (DLS) (Fig. 1c). The chelated Gd in GPT NPs is validated by the presence of the characteristic peaks corresponding to Gd 4d (142 eV) in the X-ray photoelectron spectroscopy (XPS) spectra (Fig. 1d). The presence of Gd 4d peaks indicates the oxidation state of the chelated Gd, confirming the synthesis approach is mild meanwhile the physicochemical environment for Gd coordination is still well preserved. It is noted that the XPS analysis verifies GPT NPs are composed of C, O and Gd, as evidenced by the characteristic peaks corresponding to C 1s (284 eV), O 1s (532 eV), and Gd 4d (142 eV). The results reveal the presence of the carboxyl group on the surface of GPT NPs, offering good physiological stability. Fourier Transform Infrared (FTIR) spectroscopy was applied to characterize the formation of GPT NPs. With the chelation of TCPP and presence of PEG containing groups, there were lots of surface amines and carboxyl groups on the surface of GPT NPs such as O–H (3432 cm^− 1^), N–H (2886 cm^− 1^), C=C (1700 cm^− 1^), and C–O (1112 cm^− 1^) (Additional file 1: Fig. S2). In addition, fluorescence spectroscopy analysis also confirms the presence of TCPP in GPT NPs (Additional file 1: Fig. S3). Free Gd^3+^ ions can hardly leak from GPT NPs, because TCPP is an excellent chelator for metal ions. These results revealed that only a negligible number of free Gd^3+^ ions were released from GPT NPs (Additional file 1: Fig. S4).

### In vitro T_1_-weighted MR imaging performance

**Fig. 2:**
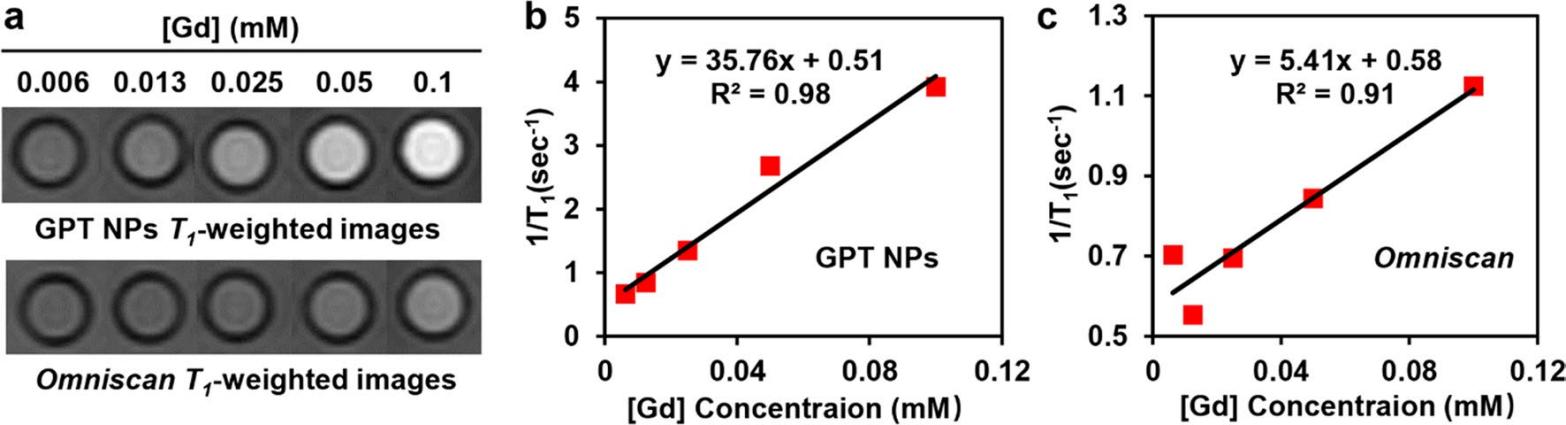
In vitro *T*_*1*_-weighted MR imaging performance. **a** In vitro* T*_*1*_-weighted MR imaging of GPT NPs and *Omniscan* at different concentrations of Gd. *T*_*1*_ relaxivity of **b** GPT NPs and **c** *Omniscan*

To estimate the performance of GPT NPs as *T*_*1*_*-*weighted contrast agents, the *r*_*1*_ value was determined by taking the slope of the linear plot of *1/T*_*1*_ versus Gd concentration. GPT NPs exhibit the contrast enhancement in *T*_*1*_*-*weighted MR images (Fig. 2a), with *r*_*1*_ value of 35.76 mM^− 1^ s^− 1^ at 3.0 T, indicating a significant increase as compared to that of commercial Gd contrast agents *Omniscan* (5.41 mM^− 1^ s^− 1^) (Fig. 2b, c). Owing to the high content of chelated Gd^3+^ with the TCPP and the direct interactions between Gd^3+^ and hydrogen protons, the *r*_*1*_ value of GPT NPs is much higher than that of clinical used Gd-based contrast agents such as *Omniscan* (3.3 mM^− 1^ s^− 1^) [19], *Magnevist* (4.1 mM^− 1^ s^− 1^) [20], *ProHance* (4.3 mM^− 1^ s^− 1^) [21], and *Gadovist* (4.34 mM^− 1^ s^− 1^) [22]. These results further demonstrate that GPT NPs have significant potential for excellent *T*_*1*_*-*contrast agents for MRA contrast enhancement.

### MR performance in vivo

**Fig. 3:**
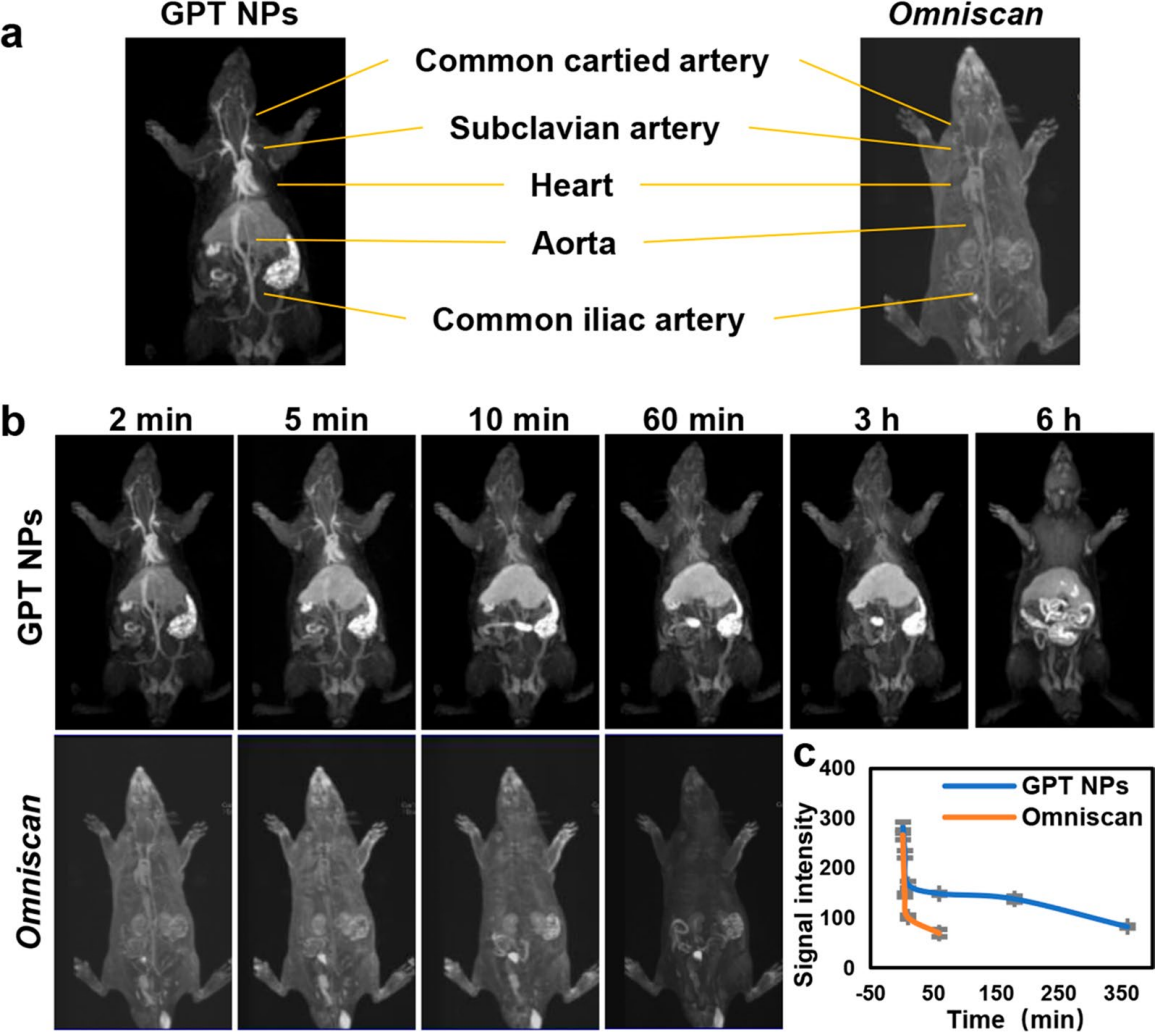
Vascular imaging performance in rats. **a** Whole body MRA images of Sprague-Dawley rats after intravenous injection of GPT NPs (left) and *Omniscan* (right). **b** Coronal section of *T*_*1*_-weighted MR images of injection of GPT NPs (upper) and *Omniscan* (down) at given time points, and the corresponding **c** MR-signal intensities of the aorta with the prolonging of duration after the intravenous administration

To investigate the performance of GPT NPs-enhanced MRA, biocompatible GPT NPs were intravenously (i.v.) injected into Sprague-Dawley rats for vascular imaging under a 3.0 T clinical MRI scanner. Immediately, the common cartied artery, subclavian artery, heart, aorta, and common iliac artery can be clearly differentiated by GPT NPs-enhanced MRA, whereas *Omniscan*-enhanced MRA images exhibit weak contrast enhancement post-injection (Fig. 3a, b). Remarkably, as revealed by the MR-signal intensities images, the signal intensity value of aorta by GPT NPs with a prolonged time window is much higher than that of using *Omniscan*, demonstrating the superior contrast effect of GPT NPs for long scan time and arterial vascular anatomy. Moreover, as revealed by the prolonging of duration after the intravenous administration of MRA images, GPT NPs were retained in blood with prolonged vascular enhancement (360 min) as compared to *Omniscan* (60 min), because of the long blood half-life of GPT NPs (Fig. 3b, c). Although the dosage of GPT NPs was 0.1 mmol kg^− 1^ of Gd as the same as the commercial *T*_*1*_ contrast agent *Omniscan*, arterial vessels of GPT NPs were substantially brightened, while contrast enhancement in the surrounding tissue was negligible. GPT NPs allowed rapid imaging of purely arterial image and the minimization of overlap with enhancing veins and tissue. On the contrary, a comparative study using *Omniscan* exhibited weak contrast in the whole-body vessels, which rendered it difficult to receive detailed vascular diagnostic information by a single injection. The MRA performance of GPT NPs showed superior vascular imaging quality and acceptance of high-spatial-resolution. The blood circulation curve illustrated that the pharmacokinetics of GPT NPs followed a two-compartment model with the half-time (T_1/2_) of 6.007 h (Additional file 1: Fig. S5). The negligible free Gd^3+^ leakage from GPT NPs by measuring the Gd concentration in blood of health rats at varied time intervals (Additional file 1: Fig. S6). These results demonstrated that GPT NPs were biocompatible with low cytotoxicity.

**Fig. 4:**
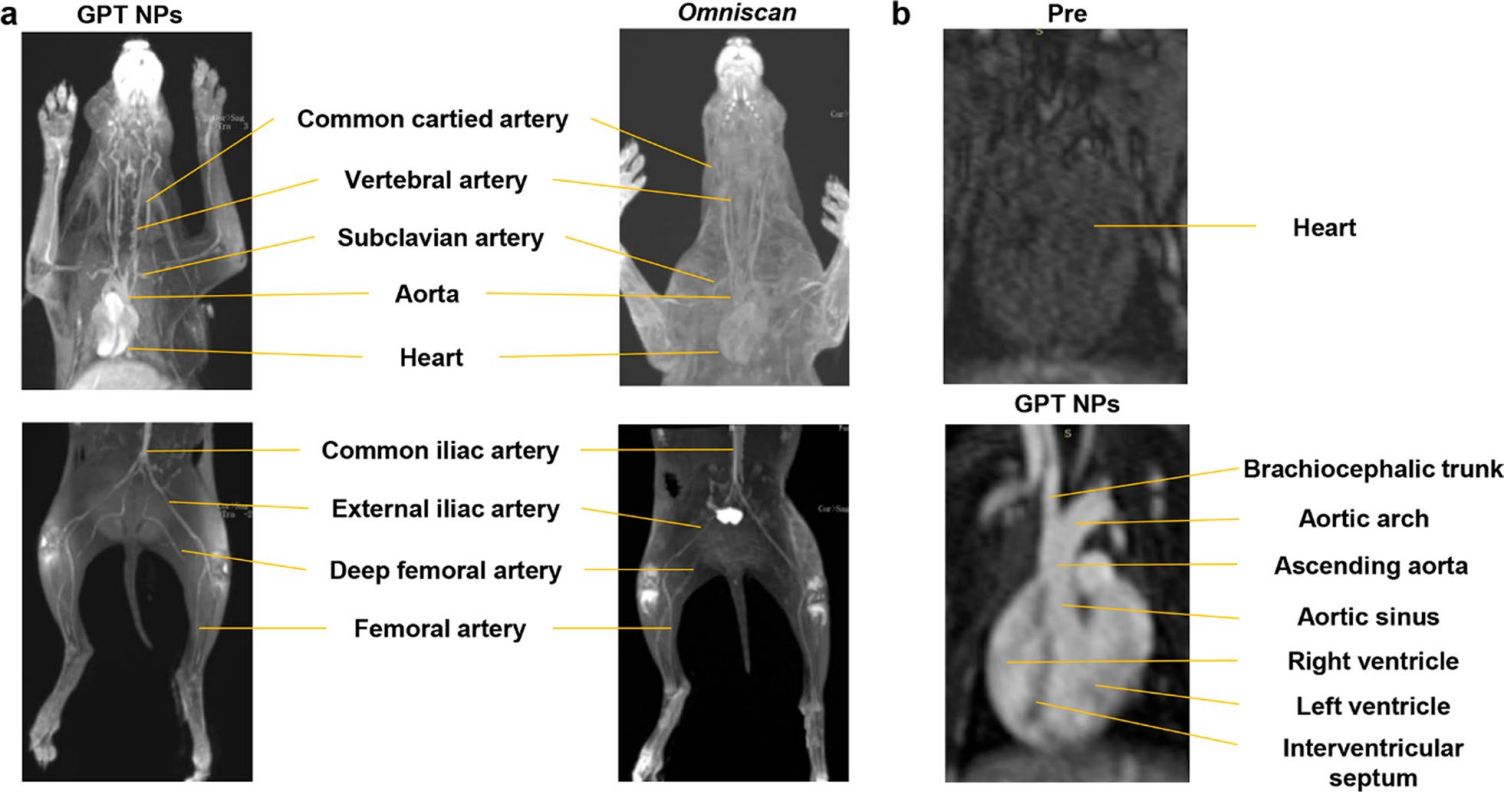
Vascular imaging performance in rabbits. **a** GPT NPs and *Omniscan* enhanced MRA images of the upper-extremity and lower-extremity vessels of New Zealand rabbits. Upper-extremity vessels, including the common cartied artery, vertebral artery, subclavian artery, aorta, and heart. Lower-extremity vessels, including the common iliac, external iliac, deep femoral, and femoral artery. **b** MRA images of the heart before and after intravenous injection of GPT NPs

Inspired by the desirable rats’ vascular performance, GPT NPs-enhanced MRA images were further conducted on larger animals (New Zealand rabbit model). GPT NPs exhibited excellent MRA performance in the visualization of upper-extremity vessels, including the common cartied artery, vertebral artery, subclavian artery, aorta, and heart. GPT NPs also enable clear visualization of lower-extremity vessels, including the common iliac artery, external iliac artery, deep femoral artery, and femoral artery. Under identical experimental conditions, MRA performance GPT NPs was superior to *Omniscan*, possibly due to its long-circulating and excellent signal-to-noise (Fig. 4a).

Heart is one of the most important organs of circulation that pumps blood to the vascular system. Cardiovascular magnetic resonance imaging has emerged as an indispensable non-invasively method to discern abnormal cardiovascular disease and cardiomyopathies [23]. However, the technique has been limited due to difficulties generated by standard extracellular contrast agents resulting in short circulating time and rapid background signal, which hampers the high-resolution MRA. Thus, the development of novel contrast agents that provide prolonged vascular enhancement and highly efficient MRA is very meaningful for clinical application. Herein, cardiovascular magnetic resonance imaging was further examined with intravenous injection of GPT NPs. Without the GPT NPs-assisted MRA, the cardiac structure is hardly observable (Fig. 4b upper). After the injection of GPT NPs, there is an immediate increase in signal intensity of the heart, including the brachiocephalic trunk, aortic arch, ascending aorta, aortic sinus, right ventricle, left ventricle, and interventricular septum (Fig. 4b lower), demonstrating the superior contrast effect of GPT NPs for assessing the cardiac vascular anatomy.

Motivated by the superior relaxivity and high SNR, biocompatible GPT NPs were injected (i.v.) into the mice bearing the subcutaneous PANC-1 pancreatic tumors and performed *T*_*1*_-weighted MRI. *Omniscan* with the equivalent concentration of Gd were used as controls. MR images at different time points before and after administration of these contrast agents are shown in Fig. S7. For a fair comparison, the two groups’ MRI signals of tumor sites were compared at given time points. There were significant enhancement signals in the GPT NPs group, compared with the *Omniscan* treated mice. It is obvious that the *T*_*1*_ contrast in the tumor sites is the strongest at 30 min for GPT NPs or 5 min post-injection for *Omniscan*, respectively (Additional file 1: Fig. S8). In addition, the MRI signal of GPT NPs at 4 h post-injection is still much stronger than that of the *Omniscan* at 5 min post-injection due to the long-time blood circulation time and super high *r*_*1*_ value.

### Biocompatibility and biosafety

An ideal MR contrast agents need to exhibit biocompatibility and biosafety. In general, free Gd^3+^ ions are considered to be cytotoxic. Therefore, it is necessary to investigate the cytotoxic effect of GPT NPs in the physiological environment. The cytotoxicity of GPT NPs and *Omniscan *in vitro was determined by the cell counting kit-8 (CCK-8) assay on pancreatic ductal epithelial cells (PDEC) and human pancreatic cancer PANC-1 cells. These cells were incubated with GPT NPs and *Omniscan* at elevated concentrations (0, 0.125, 0.25, 0.5, 1 mM) for 24 h and no obvious cytotoxicity was observed even at a concentration as high as 1 mM, demonstrating the low cytotoxicity and excellent biocompatibility of GPT NPs (Additional file 1: Figs. S9a and S10a). Then PDEC cells were incubated with GPT NPs time as long as 48 h. As expected, with the prolonged time, both GPT NPs and *Omniscan* showed negligible cytotoxicity with over 90% cell viability (Additional file 1: Fig. S9b and Sb). The relatively lower cytotoxicity of GPT NPs can be ascribed from the stabilization of Gd-chelates, which Gd^3+^ ion is very hard to be released from the nanoparticles.

To further evaluate the long-term in vivo toxicity and biocompatibility of GPT NPs, body-weight changes, hematological assessments, and H&E staining were systematically performed. Healthy BALB/c mice were i.v. injection of GPT NPs at elevated doses (0, 5, 10, 20 mg kg^− 1^) and then fed for 1-month period. No significant difference in body weight was observed compared to the control group, demonstrating the low toxicity of GPT NPs under different concentrations (Additional file 1: Fig. S11). The blood biochemistry of mice, including liver and kidney function after the i.v. administration of GPT NPs, further confirmed the biosafety of GPT NPs. Additional file 1: Fig. S12 shows no obvious hepatic toxicity (by measuring the serum levels of ALT, AST, and ALP) and Additional file 1: Fig. S13 shows no obvious kidney toxicity (by measuring the serum levels of BUN and CR) among the control group and the treatment groups. For hematological analyses, including the indexes of white blood cells analysis (Additional file 1: Fig. S14), platelets analysis (Additional file 1: Fig. S15), hemoglobin and red blood cells analysis (Additional file 1: Fig. S16, S17), all measured indicators appeared to be normal compared to those in the control group. Histological analyses of the main organs (heart, liver, spleen, lung, and kidney) in all groups were performed by hematoxylin and eosin (H&E) staining, and no significant tissue abnormalities or severe inflammation were detected in these tissue (Additional file 1: Fig. S18). All aforementioned biocompatibility analysis results elucidates the low toxicity of GPT NPs under administration dose. The GPT NPs are intrinsically featured with good biocompatibility for potential clinical translation, especially for further *T*_*1*_-weighted MRA imaging.

## Conclusions

In summary, we successfully developed novel Gd-chelated PEG-TCPP nanoparticles (GPT NPs) with super high *r*_*1*_ relaxivity and low free Gd^3+^ leaking for *T*_*1*_ contrast-enhanced MRA. Owing to the high content of chelated Gd^3+^ with TCPP and compact PEG modifications, GPT NPs not only amplify the *T*_*1*_ contrast ability, but also prolong the circulation time. GPT NPs exhibit a high relaxation rate of 35.76 mM^− 1^ s^− 1^, which is much higher than the most reported *T*_*1*_-weighted MR contrast agents, demonstrating that GPT NPs can be used as efficient *T*_*1*_ contrast agents. Especially, by virtue of high *r*_*1*_ relaxivity, long blood circulation time, and low toxicity, GPT NPs are capable of excellent contrast between arteries and surrounding veins, and minimization of overlap with enhancing veins and tissue. Moreover, GPT NPs also show excellent details in cardiac vascular anatomy as well as extend circulation time in blood. It is postulated that GPT NPs have potential application in accurately medical diagnosis of vascular related diseases owing to their extreme high-resolution and biocompatibility.

## Supplementary Information

**Additional file 1: Section S1.** In vivo toxicity experiments. **Fig. S1.** Negative-staining TEM image of the GPT NPs. **Fig. S2.** Fourier Transform Infrared (FTIR) spectroscopy of the GPT NPs. **Fig. S3.** Fluorescence spectroscopy of GPT NPs. **Fig. S4.** The free Gd^3+^ leakage from GPT NPs and *Omniscan*. **Fig. S5.** Blood circulation curve of GPT NPs. **Fig. S6.** The free Gd^3+^ leakage from GPT NPs. **Fig. S7.** *T*_1_-weighted MRA images of PANC-1 tumor-bearing mice before and after intravenous GPT NPs and *Omniscan*. **Fig. S8.** *T*_1_-weighted MRI-signal intensities of tumor site after the intravenous administration of GPT NPs and *Omniscan*. **Fig. S9.** Relative viabilities of PDEC cells and PANC-1 cancer cells after incubation with GPT NPs. **Fig. S10.** Relative viabilities of PDEC cells and PANC-1 cancer cells after incubation with *Omniscan*. **Fig. S11.** Time-dependent body-weight changing profiles of BALB/c mice after intravenous administration of GPT NPs. **Fig. S12.** Liver function indexes of BALB/c mice after intravenous injection of GPT NPs. **Fig. S13.** Kidney function indexes of BALB/c mice after intravenous injection of GPT NPs. **Fig. S14.** Hematological white blood cells analyses of BALB/c mice after intravenous injection of GPT NPs. **Fig. S15.** Hematological platelets analyses of BALB/c mice after intravenous injection of GPT NPs. **Fig. S16–S17.** Hematological hemoglobin and red blood cells analyses of BALB/c mice after intravenous injection of GPT NPs. **Fig. S18.** H&E-stained tissues sections from BALB/c mice of BALB/c mice after intravenous injection of GPT NPs.

## Data Availability

All study data are included in this article.
