# Supplementary material for: High relaxivity Gd3+-based organic nanoparticles for efficient magnetic resonance angiography
Source: J Nanobiotechnology. 2022 Mar 31;20:170. doi: 10.1186/s12951-022-01363-3 (PMC8973627; doi:10.1186/s12951-022-01363-3)
Supplement: Supplementary file 1 — Additional file 1: Section S1. In vivo toxicity experiments. Fig. S1. Negative-staining TEM image of the GPT NPs. Fig. S2. Fourier Transform Infrared (FTIR) spectroscopy of the GPT NPs. Fig. S3. Fluorescence spectroscopy of GPT NPs. Fig. S4. The free Gd3+ leakage from GPT NPs and Omniscan. Fig. S5. Blood circulation curve of GPT NPs. Fig. S6. The free Gd3+ leakage from GPT NPs. Fig. S7. T1-weighted MRA images of PANC-1 tumor-bearing mice before and after intravenous GPT NPs and Omniscan. Fig. S8. T1-weighted MRI-signal intensities of tumor site after the intravenous administration of GPT NPs and Omniscan. Fig. S9. Relative viabilities of PDEC cells and PANC-1 cancer cells after incubation with GPT NPs. Fig. S10. Relative viabilities of PDEC cells and PANC-1 cancer cells after incubation with Omniscan. Fig. S11. Time-dependent body-weight changing profiles of BALB/c mice after intravenous administration of GPT NPs. Fig. S12. Liver function indexes of BALB/c mice after intravenous injection of GPT NPs. Fig. S13. Kidney function indexes of BALB/c mice after intravenous injection of GPT NPs. Fig. S14. Hematological white blood cells analyses of BALB/c mice after intravenous injection of GPT NPs. Fig. S15. Hematological platelets analyses of BALB/c mice after intravenous injection of GPT NPs. Fig. S16–S17. Hematological hemoglobin and red blood cells analyses of BALB/c mice after intravenous injection of GPT NPs. Fig. S18. H&E-stained tissues sections from BALB/c mice of BALB/c mice after intravenous injection of GPT NPs. [file 12951_2022_1363_MOESM1_ESM.docx]

Supplementary Material

High relaxivity Gd^3+^-based organic nanoparticles for efficient magnetic resonance angiography

Zhuang Liu^1†^, Menglong Zhao^2†^, Han Wang^3†^, Zi Fu^1,3^, Hongbo Gao^4^, Weijun Peng^1^, Dalong Ni^3*^, Wei Tang^1*^, Yajia Gu^1*^

^1^ *Department of Radiology, Fudan University Shanghai Cancer Center**, Department of Oncology, Shanghai Medical College, Fudan University, Shanghai, 200032, China*

^2^ *Department of Radiology, Zhongshan Hospital, Fudan University and Shanghai Institute of Medical Imaging, Shanghai, 200032, China*

^3^ *Department of Orthopaedics, Shanghai Key Laboratory for Prevention and Treatment of Bone and Joint Diseases, Shanghai Institute of Traumatology and Orthopaedics, Ruijin Hospital, Shanghai Jiao Tong University School of Medicine, Shanghai 200025, China*

^4^ *Department of Radiation Oncology, Huadong Hospital Affiliated to Fudan University, Shanghai 200040, China*

^†^Zhuang Liu, Menglong Zhao and Han Wang contributed equally to this work.

*Corresponding: ndl12353@rjh.com.cn; tangwei105@163.com; cjr.guyajia@vip.163.com

**Supplementary Material**

***In vivo* toxicity experiments**

All animal studies were conducted with the approval and according to the recommendations established by the administrative committee of laboratory animals of Fudan University. Healthy male BALB/c mice (4 weeks) were purchased from Shanghai SLAC Laboratory Animal Co. Ltd and maintained in a specific pathogen-free (SPF) environment during the experiments. Sixteen BALB/c mice were randomly divided into four groups (n = 4) and intravenously administered with GPT NPs at elevated doses (0, 5, 10, 20 mg kg^-1^). During the 30 days, the body weight of mice was measured every other day. Hematological and histological analyses were performed on the 30^th^ day after intravenous injection. Liver function indexes were measured by the serum levels of alanine aminotransferase (ALT), aspartate aminotransferase (AST), alkaline phosphatase (ALP). Kidney function indexes were measured by the serum levels of blood urea nitrogen (BUN) and creatinine (CR). Hematological white blood cells analyses were measured by the serum levels of white blood cells (WBC), lymphocyte (LYM), monocyte (MON), and granulocyte (GRAN). Hematological platelets analyses were measured by the serum levels of platelets count (PLT), mean platelets volume (MPV), platelets ratio (PCT), and platelet-large cell rate (P-LCR). Hemoglobin and red blood cells analyses were measured by the serum levels of red blood cells (RBC), hemoglobin (HGB), hematocrit (HCT), mean corpuscular volume (MCV), mean corpuscular hemoglobin (MCH), mean corpuscular hemoglobin concentration (MCHC), red blood cell volume distribution width (RDW) and red blood cell volume distribution width standard deviation (RDW-SD).

**Supplementary Figures**

**Characterization of Gd-chelated PEG-TCPP nanoparticles (GPT NPs)**


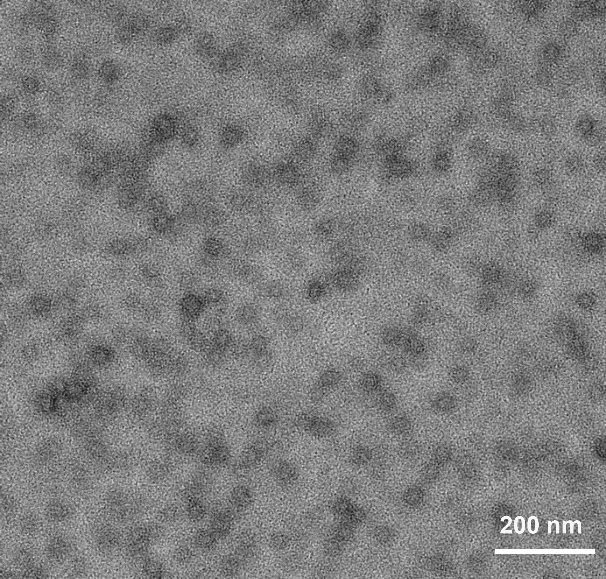


**Fig. S1** Negative-staining TEM image of the GPT NPs.


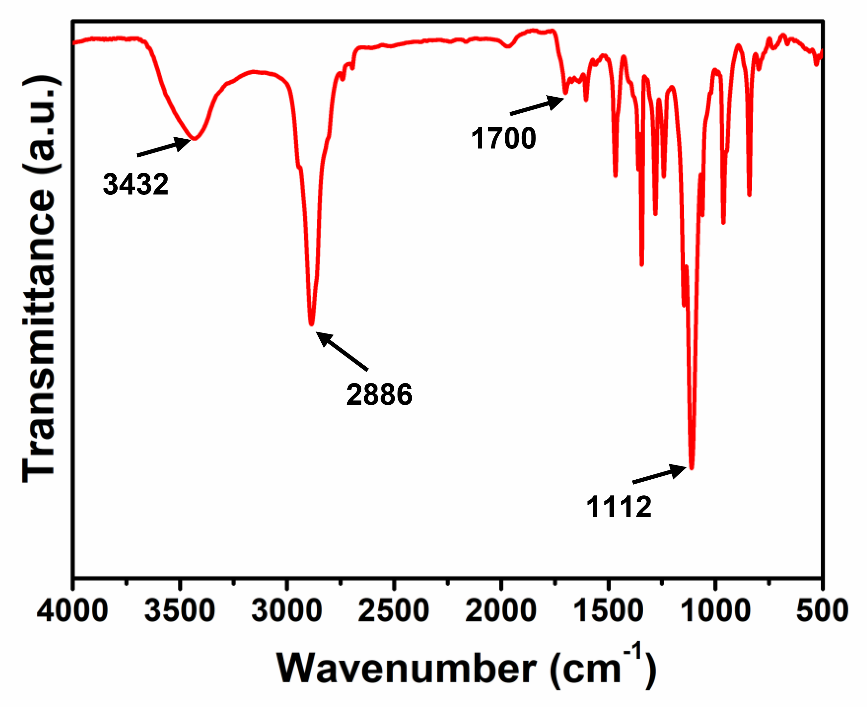


**Fig. S2** Fourier Transform Infrared (FTIR) spectroscopy of the GPT NPs.


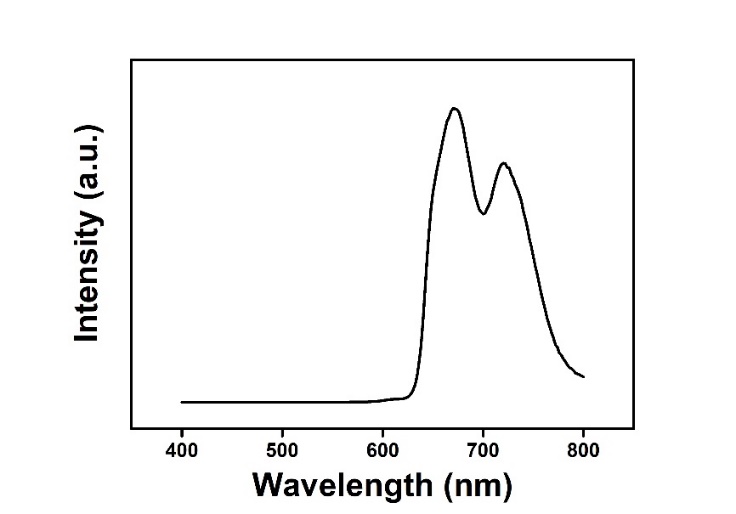


**Fig. S3** Fluorescence spectroscopy of GPT NPs.


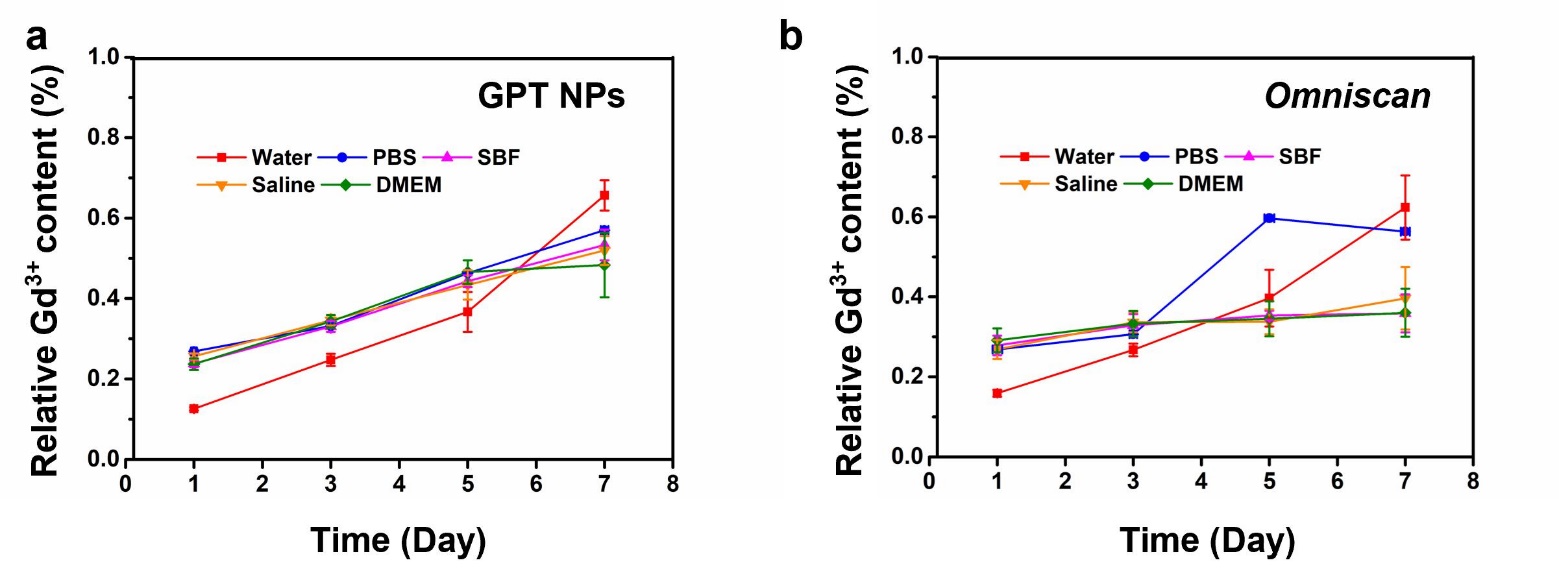


**Fig. S4** The free Gd^3+^ leakage from GPT NPs and *Omniscan* at different time points in different buffer solutions (Water, PBS, SBF, Saline, and DMEM).

**Blood circulation curve of GPT NPs**


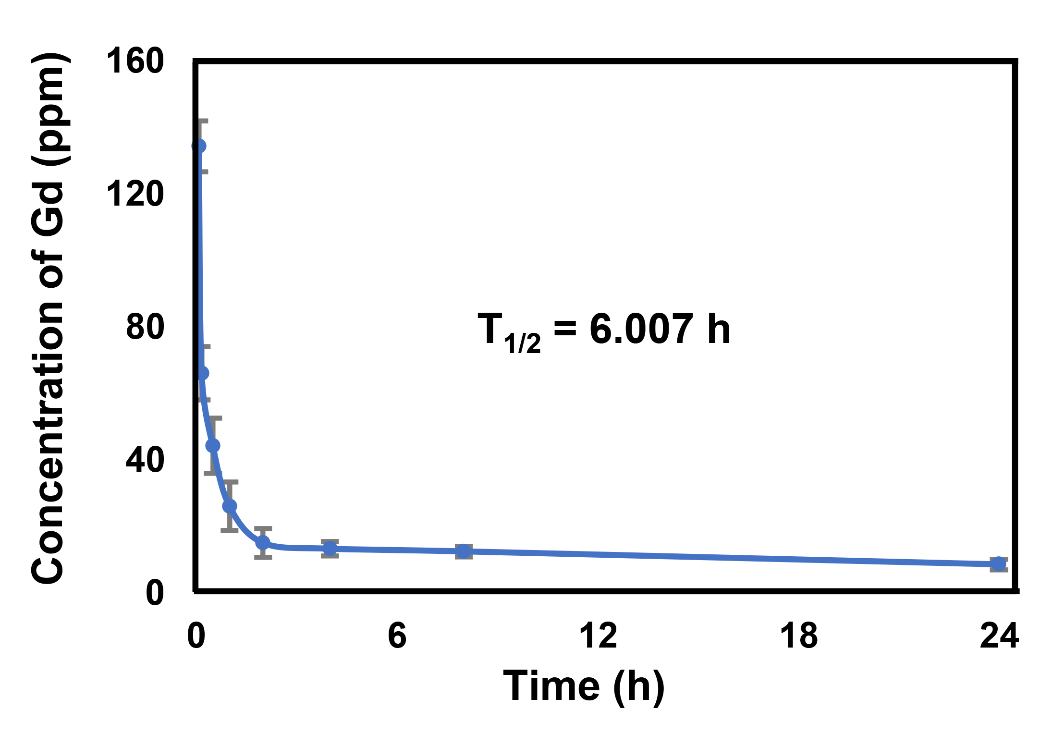


**Fig. S5** Blood circulation curve of GPT NPs by measuring the Gd concentration in blood of health rats at different time points intervals post *i.v.* injection (n = 3). The half-time (T_1/2_) is calculated to be approximately 6.007 h.

**The free Gd^3+^ leakage from GPT NPs**


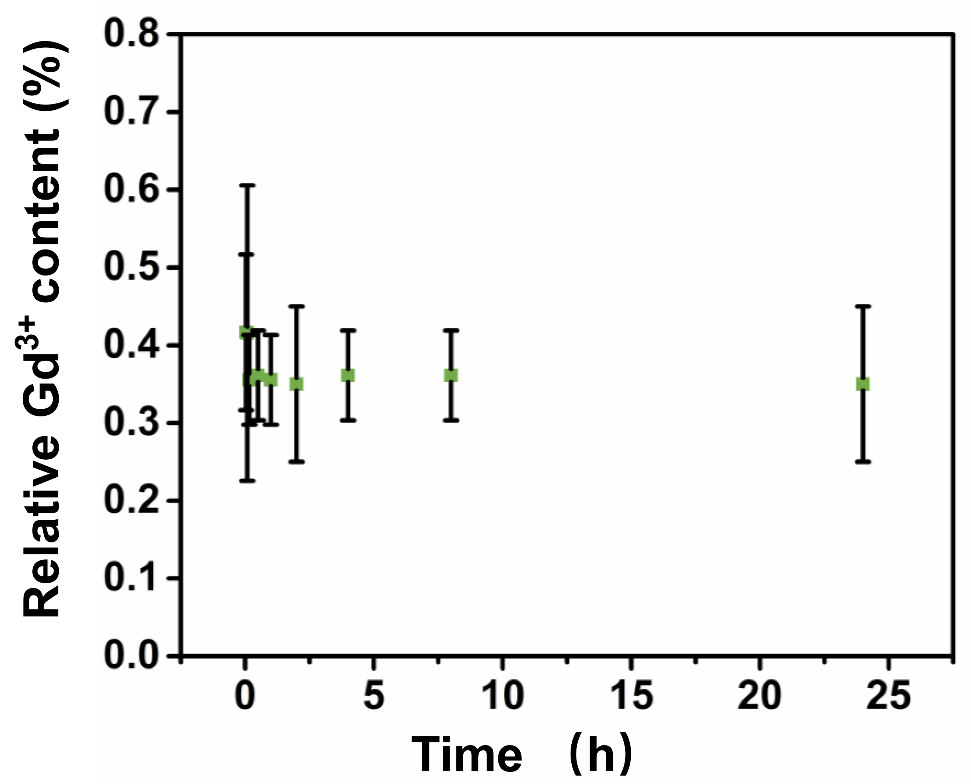


**Fig. S6** The free Gd^3+^ leakage from GPT NPs by measuring the Gd concentration in blood of health rats at varied time intervals.

**MR performance *in vivo***


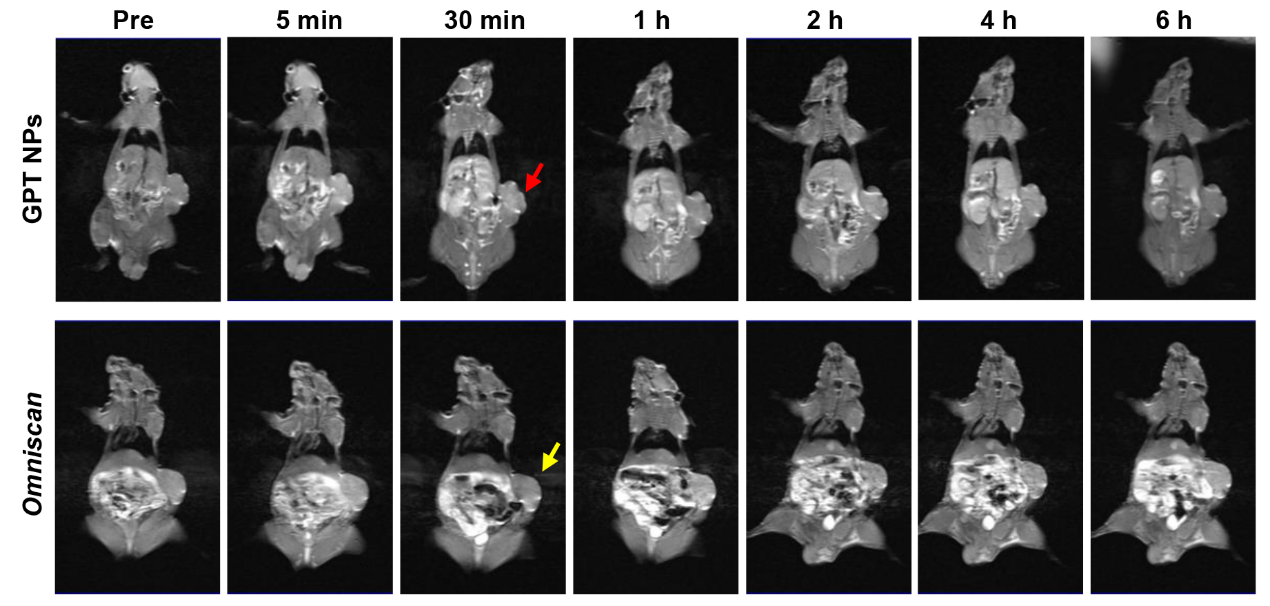


**Fig. S7** Coronal section of *T_1_*-weighted MRA images of PANC-1 tumor-bearing mice before and after intravenous GPT NPs (upper) and *Omniscan* (down) at given time points. The tumor sites were marked by red and yellow arrows.


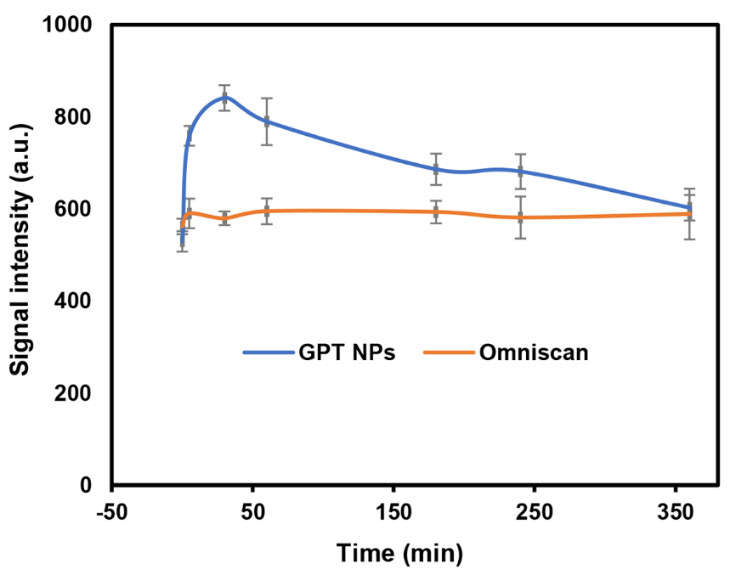


**Fig. S8** *T_1_*-weighted MRI-signal intensities of tumor site with the prolonging of duration after the intravenous administration of GPT NPs and *Omniscan*.

Biocompatibility and biosafety


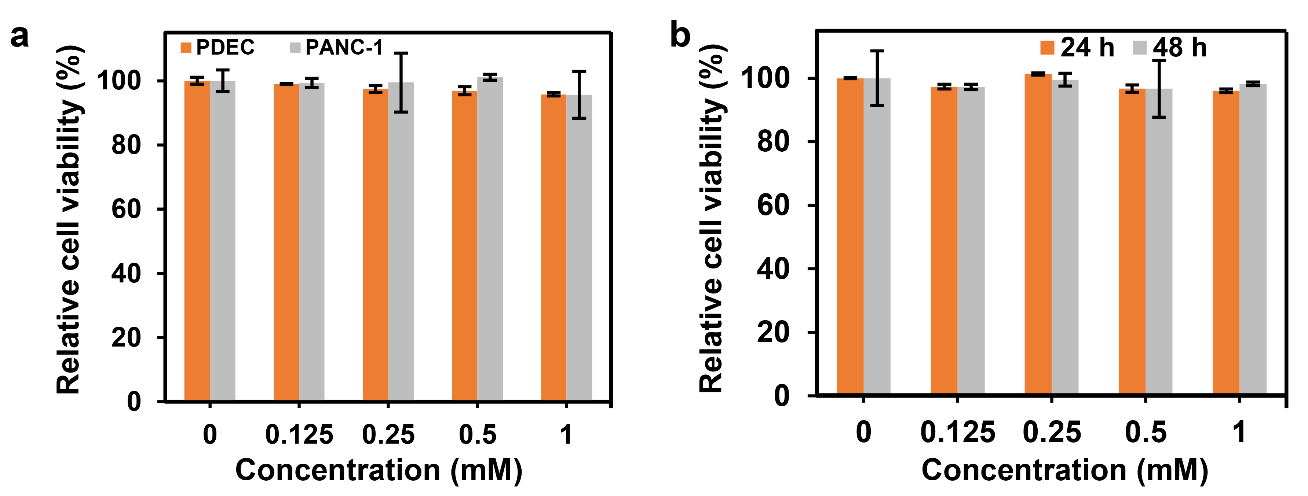


**Fig. S9** **a** Relative viabilities of PDEC cells and PANC-1 cancer cells after incubation with GPT NPs at elevated concentrations (0, 0.125, 0.25, 0.5, 1 mM) for 24 h. **b** Relative viabilities of PDEC cells after incubation with GPT NPs at elevated concentrations (0, 0.125, 0.25, 0.5, 1 mM) for 24 h and 48 h. Error bars were based on the standard deviations (SD).


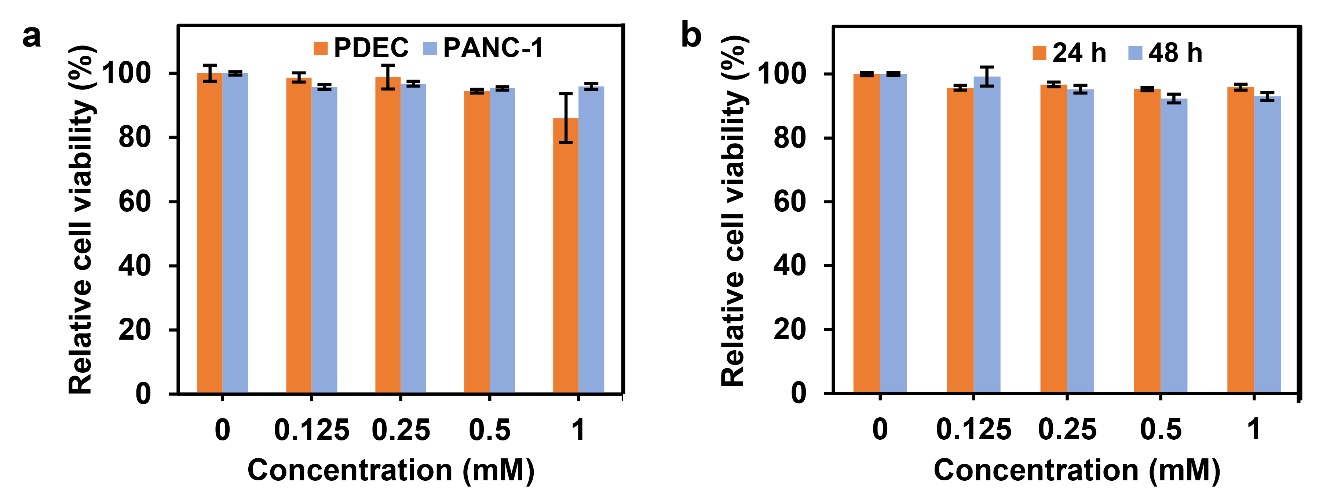


**Fig. S10** **a** Relative viabilities of PDEC cells and PANC-1 cancer cells after incubation with *Omniscan* at elevated concentrations (0, 0.125, 0.25, 0.5, 1 mM) for 24 h. **b** Relative viabilities of PDEC cells after incubation with *Omniscan* at elevated concentrations (0, 0.125, 0.25, 0.5, 1 mM) for 24 h and 48 h. Error bars were based on the standard deviations (SD).


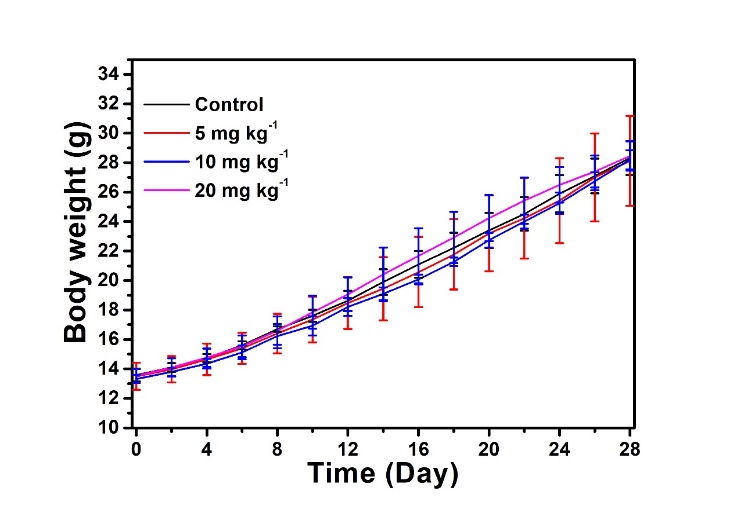


**Fig. S11** Time-dependent body-weight changing profiles of BALB/c mice within 30 days after intravenous administration of GPT NPs at elevated doses (0, 5, 10, and 20 mg kg^-1^).


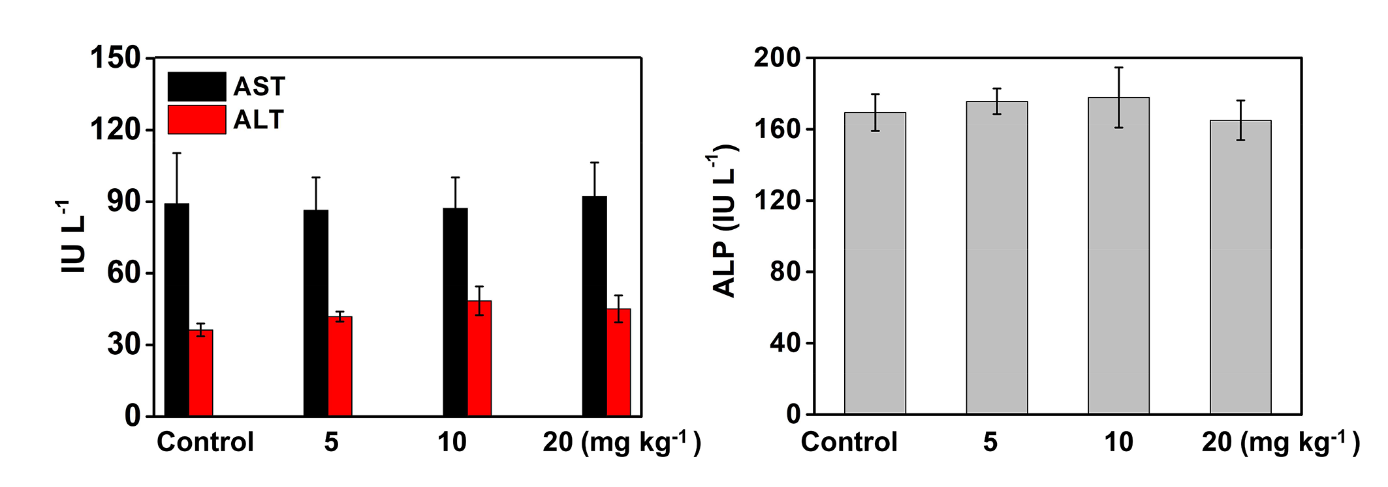


**Fig. S12** Liver function indexes (by measuring the serum levels of ALT, AST, and ALP) of BALB/c mice after single intravenous injection of GPT NPs at elevated doses (0, 5, 10, and 20 mg kg^-1^) for 30 days feeding.


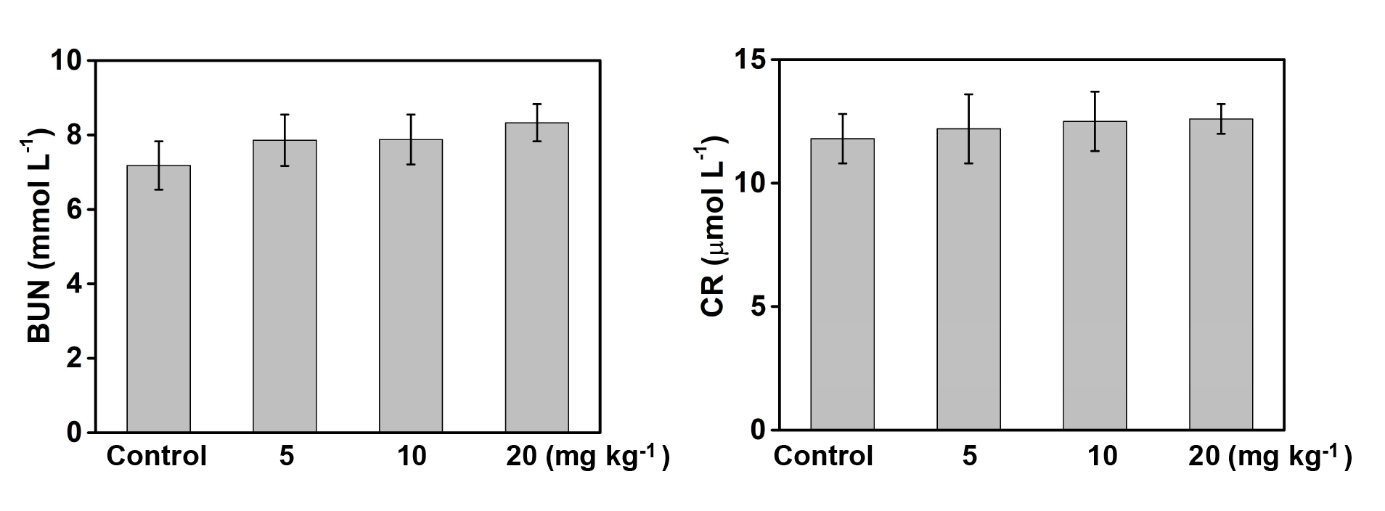


**Fig. S13** Kidney function indexes (by measuring the serum levels of BUN and CR) of BALB/c mice after single intravenous injection of GPT NPs at elevated doses (0, 5, 10, and 20 mg kg^-1^) for 30 days feeding.


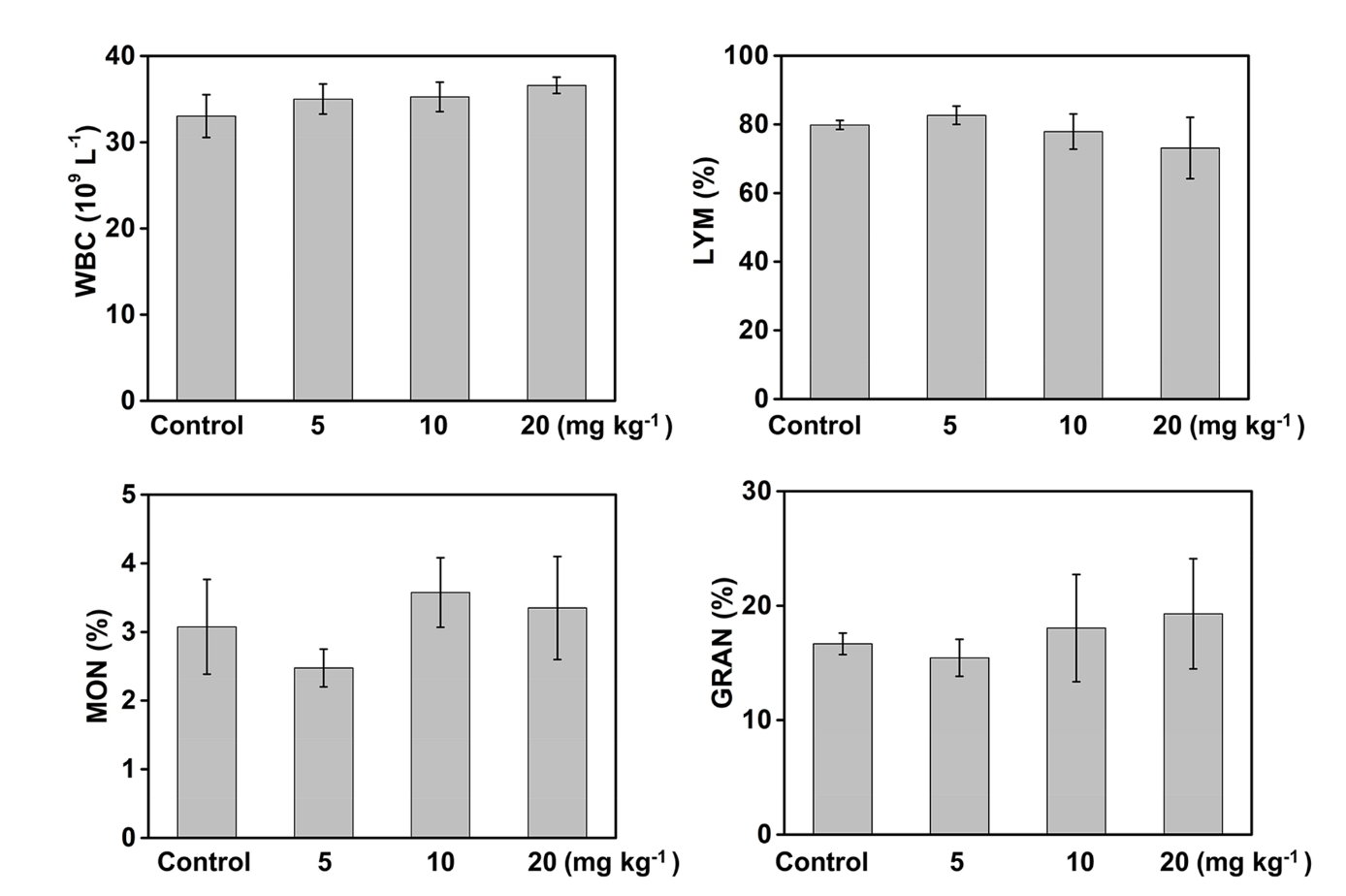


**Fig. S14** Hematological white blood cells analyses (by measuring the serum levels of WBC, LYM, MON, and GRAN) of BALB/c mice after single intravenous injection of GPT NPs at elevated doses (0, 5, 10, and 20 mg kg^-1^) for 30 days feeding.


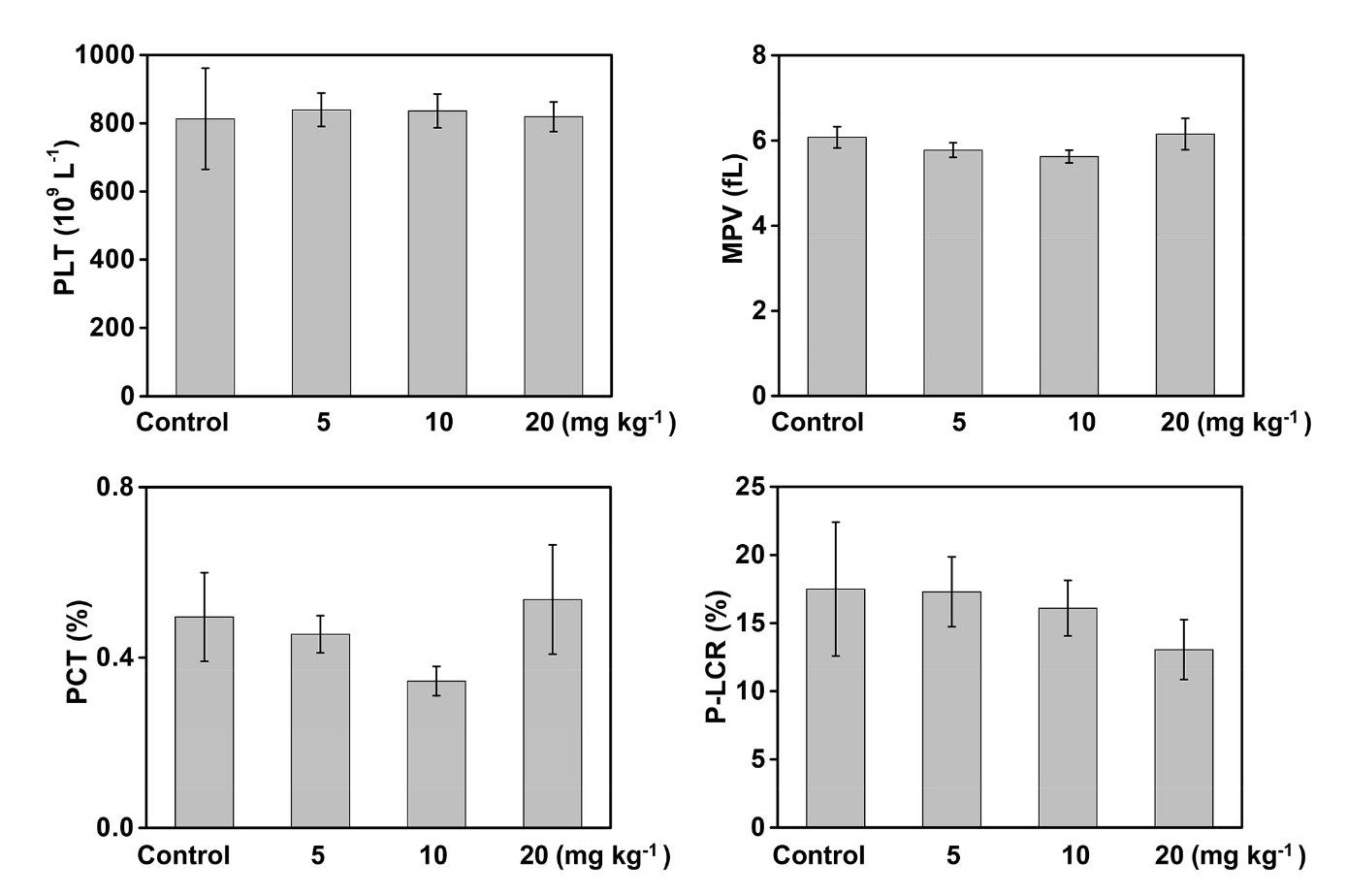


**Fig. S15** Hematological platelets analyses (by measuring the serum levels of PLT, MPV, PCT, and P-LCR) of BALB/c mice after single intravenous injection of GPT NPs at elevated doses (0, 5, 10, and 20 mg kg^-1^) for 30 days feeding.


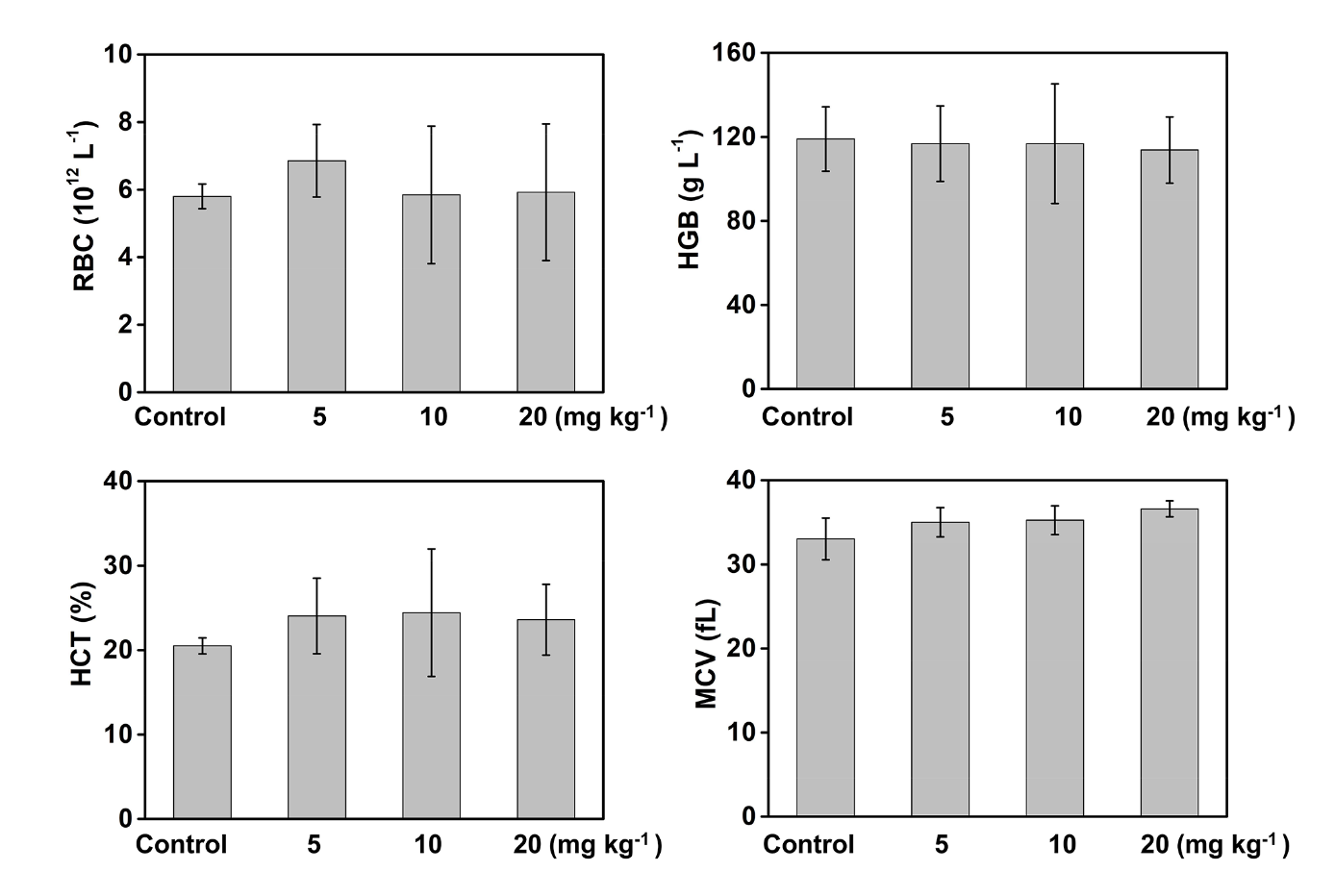


**Fig. S16** Hematological hemoglobin and red blood cells analyses (by measuring the serum levels of RBC, HGB, HCT, and MCV) of BALB/c mice after single intravenous injection of GPT NPs at elevated doses (0, 5, 10, and 20 mg kg^-1^) for 30 days feeding.


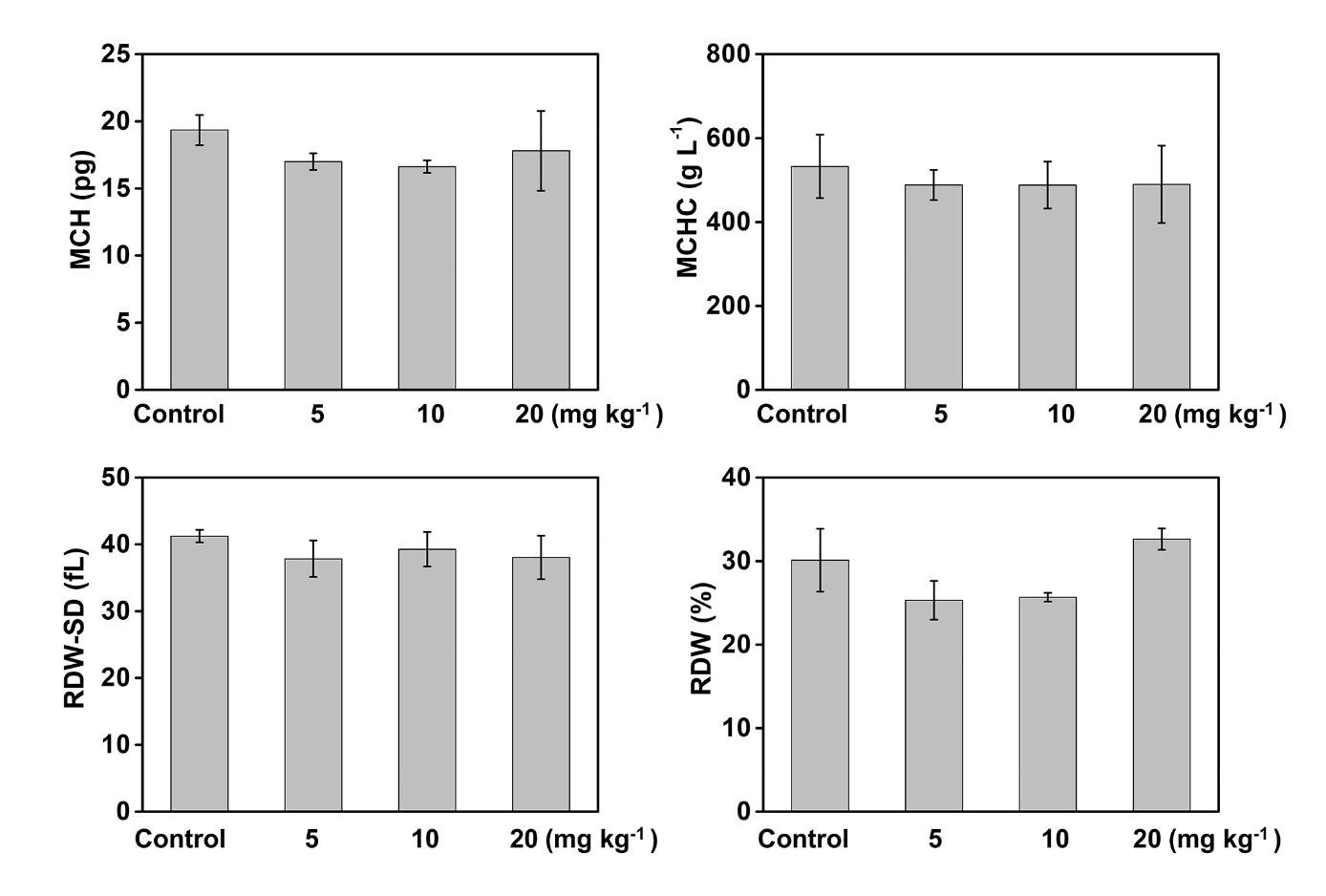


**Fig. S17** Hematological red blood cells analyses (by measuring the serum levels of MCH, MCHC, RDW-SD, and RDW) of BALB/c mice after single intravenous injection of GPT NPs at elevated doses (0, 5, 10, and 20 mg kg^-1^) for 30 days feeding.


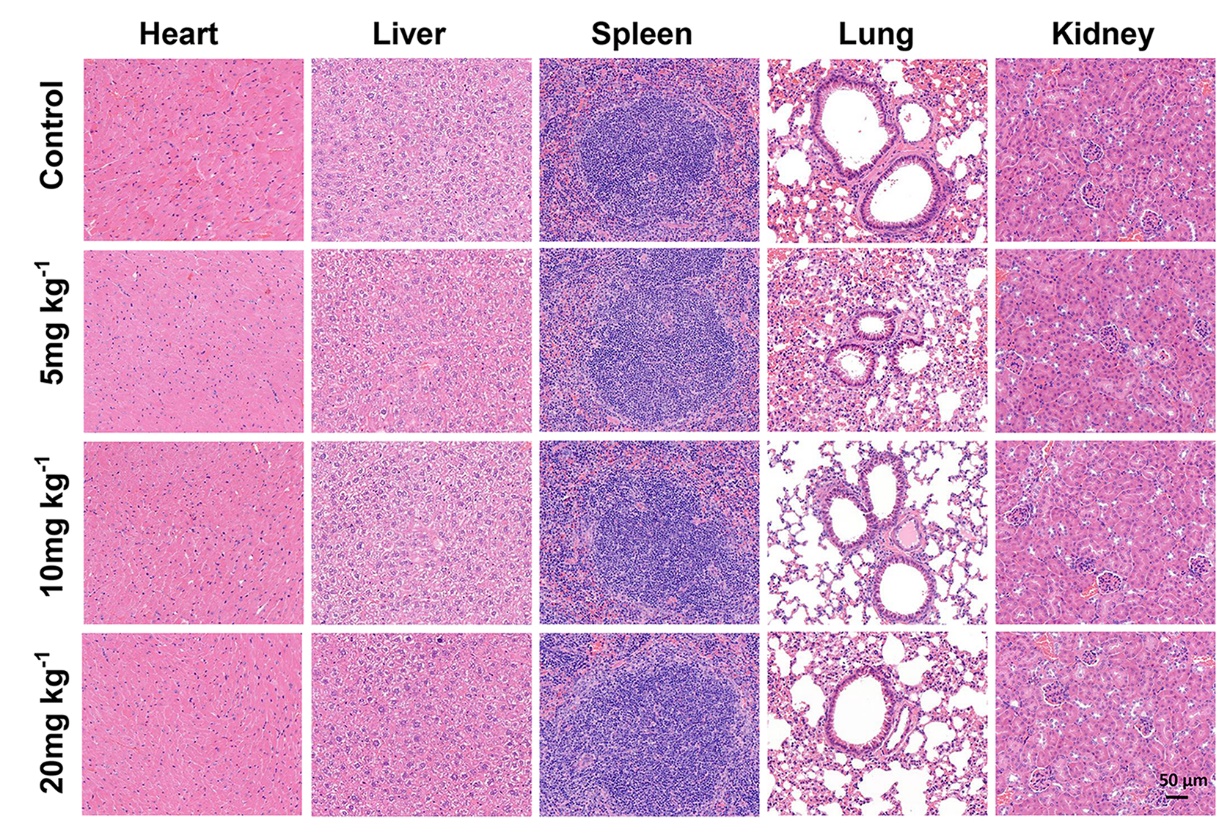


**Fig. S18** H&E-stained tissues sections from BALB/c mice to monitor the histological changes in the heart, liver, spleen, lung, and kidney at 30^th^ days after a single intravenous injection of GPT NPs (0, 5, 10, and 20 mg kg^-1^). All images share the same scale bar of 50 μm.
